# RNA Sequencing Analysis to Capture the Transcriptome Landscape during Tenderization in Sea Cucumber *Apostichopus japonicus*

**DOI:** 10.3390/molecules24050998

**Published:** 2019-03-12

**Authors:** Xiufang Dong, Hang Qi, Baoyu He, Di Jiang, Beiwei Zhu

**Affiliations:** School of Food Science and Technology, Dalian Polytechnic University, National Engineering Research Center of Seafood, Dalian 116034, China; dxf900321@126.com (X.D.); heby0805@163.com (B.H.); jiangdi96213@126.com (D.J.)

**Keywords:** sea cucumber *Apostichopus japonicus*, body wall, tenderization mechanism, RNA-seq, differentially expressed genes, oxidative stress response

## Abstract

Sea cucumber (*Apostichopus japonicus*) is an economically significant species in China having great commercial value. It is challenging to maintain the textural properties during thermal processing due to the distinctive physiochemical structure of the *A. japonicus* body wall (AJBW). In this study, the gene expression profiles associated with tenderization in AJBW were determined at 0 h (CON), 1 h (T_1h), and 3 h (T_3h) after treatment at 37 °C using Illumina HiSeq™ 4000 platform. Seven-hundred-and-twenty-one and 806 differentially expressed genes (DEGs) were identified in comparisons of T_1h vs. CON and T_3h vs. CON, respectively. Among these DEGs, we found that two endogenous proteases—72 kDa type IV collagenase and matrix metalloproteinase 16 precursor—were significantly upregulated that could directly affect the tenderness of AJBW. In addition, 92 genes controlled four types of physiological and biochemical processes such as oxidative stress response (3), immune system process (55), apoptosis (4), and reorganization of the cytoskeleton and extracellular matrix (30). Further, the RT-qPCR results confirmed the accuracy of RNA-sequencing analysis. Our results showed the dynamic changes in global gene expression during tenderization and provided a series of candidate genes that contributed to tenderization in AJBW. This can help further studies on the genetics/molecular mechanisms associated with tenderization.

## 1. Introduction

The sea cucumber (*Apostichopus japonicus*) is naturally found along the coasts of China, Japan, Korea, and Russia of Northeast Asia and intensively cultured in East Asian countries [1]. *A. japonicus* achieved the maximum single-species production value and revenue in northern China (Liaoning and Shandong provinces) with nearly 220,000 tons production [2] and estimated value of about 4 Bn USD in 2015. Traditionally, sea cucumbers are generally processed into dry products, however, in recent years new products, such as instant sea cucumbers, with more nutritional value are being developed. Nevertheless, it is difficult to maintain fine and stable texture during regular thermal processing even for instant sea cucumbers due to the distinctive physiochemical structure of *A. japonicus* body wall (AJBW). This particular problem has caused substantial economic losses in the seafood industry [3,4].

Tenderness is generally considered as the basic characteristic of meat related to mouth feeling quality. Therefore, tenderization, a common technology in meat processing, was developed to break down the collagens to obtain better palatability. It has been reported that low temperature heating was effective to improve the tenderness of beef and pork [5]. The improved tenderness of meat heated at low temperature may be attributed partially to the action of proteolytic enzymes, causing weakening of fibrils, and solubilization of collagen [6]. Similarly, our previous studies also demonstrated that the hardness and chewiness of AJBW decreased after the low temperature heating time was extended and developed a method using nuclear magnetic resonance and magnetic resonance imaging to assess the proton changes of AJBW [7,8]. Tenderization process mainly involves (1) hydrolysis of muscle tissues by the endogenous proteases, like cathepsins, calpains, and caspases [9,10,11], and (2) assessing physicochemical properties of the muscle protein [12,13]. Previous reports have demonstrated that the release and activation of endogenous enzymes could be induced by apoptosis during meat tenderization [14,15]. However, there are no reports on the potential biological processes of the activation of endogenous proteases in *A. japonicus* during tenderization process.

The transcriptome is a set of all RNA transcripts and its modifications can exert its effect of protein translation on the phenotype of the organism [16]. Therefore, transcriptome analysis is essential for elucidating the underlying molecular constituents of cells and tissues in various biological processes. In this context, RNA-Seq technology has been widely applied in histological analysis [17], immunology [18], physiology [19], embryonic development, and gene markers [20] in *A. japonicus*. In addition, it has been used to investigate the effect of heat stress on marine animals such as rainbow trout [21], abalone [22], coral [23], and zooplankton [24]. These published articles found that heat shock could induce oxidative stress, immune response, and protein degradation, thus explaining the mechanism of the heat tolerance. 

In sea cucumber, endogenous proteases, like cysteine proteinases [25] and matrix metalloproteinases [26], have been found to degrade the protein in the body wall, while their gene expressions were regulated by some inflammatory cytokines such as tumor necrosis factor (TNF) and interleukin-1 (IL-1) [27]. In this study, the transcriptome of AJBW during tenderization was determined using Illumina Hiseq 4000 and comparative analysis was performed to understand (1) the type of endogenous protease involved and (2) the physiological and biochemical processes that were induced at the mRNA levels of AJBW during tenderization. These new findings would help to understand the tenderization mechanism of AJBW and provide a theoretical basis for the determination of suitable thermal processing conditions.

## 2. Results

### 2.1. Transcriptome Sequencing and Quality Control

In this study, nine cDNA libraries were constructed from AJBW following the treatment at different times and sequenced to obtain a comprehensive analysis of tenderization progression. Totals of 48,381,506–58,609,160 raw reads were generated in each library, and the clean reads were 47,514,898–57,471,652 reads with valid ratios of over 97%. Q20 > 99% and Q30 > 91% in each library suggested that the quality of the data was superior (Table 1). In addition, the evenly distributed reads in every position of the genes indicated that the randomness of the breaking was fair. However, we found that the sample correlations were more than 0.6 except in the T_1h_1 samples (the least one was 0.394, Supplementary Figure S1). Thus, the cDNA library of T_1h_1 was excluded to ensure the results were consistent and practical for further analysis of differentially-expressed genes (DEGs).

### 2.2. Reads Mapping to the Reference Genome Dataset

All the clean reads were mapped to the reference genes expressed in the *A. japonicus* genome [28]. We found that 70.44–82.03% of the reads were perfectly matched to the reference genome in each library. In unique mapped reads, 39.54–44.52% (18,785,041–24,024,127) and, in multimapped reads, 28.73–43.71% (16,510,649–21,860,562) was matched (Table 2). Gene expression of each library showed a normal distribution (Supplementary Figure S2), indicating consistency in the gene expression of three biological replicates. Meanwhile, most reads matched with exon regions from each library (Supplementary Figure S3).

In total, 52,878 genes were detected in three stages (CON, T_1h, and T_3h) in the AJBW through RNA-seq analysis. Among them, 57.40% (30,350 genes) were mapped to the reference genes with homologous sequences in at least one of the databases (Supplementary Table S1). Moreover, 5415 (17.84%) showed significant matches to Gene Ontology (GO); 3301 (10.88%) to Kyoto Encyclopedia of Genes and Genomes (KEGG); 19,149 (63.09%) to NCBI nonredundant protein sequences (Nr); 12,834 (42.29%) to Swissprot; and 14,875 (49.01%) to Protein family (Pfam). In short, approximately 69.57% (21,115 genes) of the total annotated genes from the AJBW were noted by the main five common databases.

### 2.3. Analysis of DEGs

**Figure 1:**
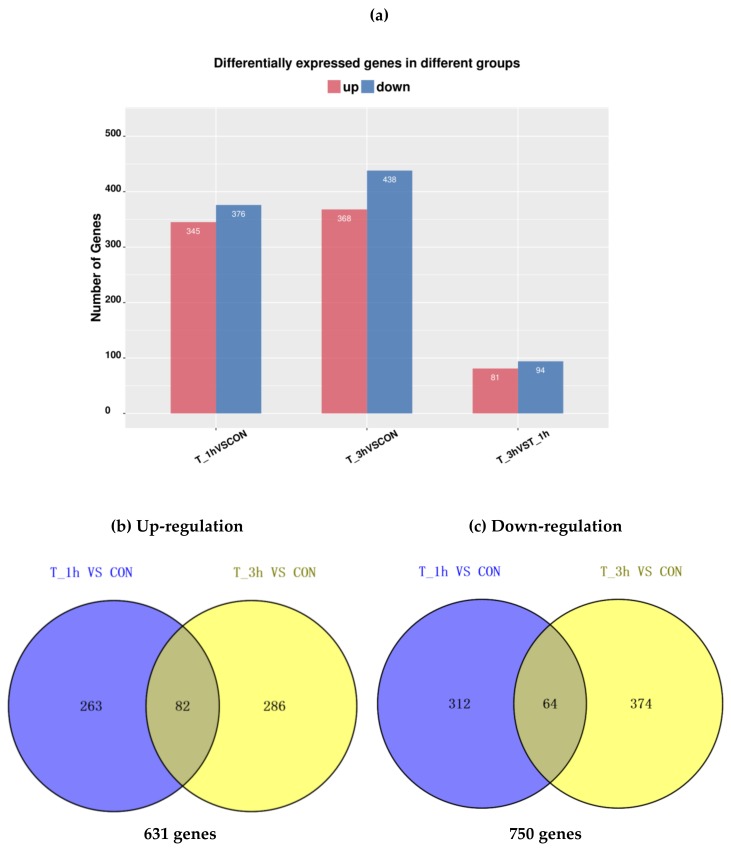
(**a**) The differentially-expressed genes (DEGs) of AJBW during tenderization. (**b**) Upregulated genes of T_1h and T_3h compared to CON. (**c**) Downregulated genes of T_1h and T_3h compared to CON.

Compared to the fresh AJBW (CON), 721 and 806 significant DEGs (|log2Ratio| ≥ 1 and *p* ≤ 0.05) were identified in T_1h and T_3h, respectively (Figure 1a). Out of these DEGs, 345 upregulated genes and 376 downregulated genes were identified from the comparison of T_1h vs. CON; while 368 upregulated and 438 downregulated were screened from the comparison of T_3h vs. CON. In addition, a strategy was developed to focus on key tenderization genes by comparing the gene expression between T_3h and T_1h. Consequently, only 81 upregulated genes and 94 downregulated genes were screened from the comparison of T_3h vs. T_1h.

At the two time points studied, after treatment, the total downregulated genes (750) was slightly more than upregulated genes (631) (Figure 1b,c). There were 146 DEGs consistently upregulated or downregulated in T_1h and T_3h. These upregulated or downregulated DEGs with annotation are presented in Supplementary Table S2. The consistent DEGs contained some important extracellular matrix (ECM)-associated genes (7), such as alpha-2 collagen, 72 kDa type IV collagenase, and matrix metalloproteinase 16 precursor; five cytoskeletal genes, such as myosin heavy chain, troponin I, and titin; and six immune-related genes, such as lipo-polysaccharide (LPS)-induced TNF-alpha factor, fibrinogen-like protein A, complement component 3 C3 (C3), and complement factor B (Bf).

Based on the primary results, the main DEGs that might exhibit important functions when AJBW was tenderized are shown in Table 3. (The total 94 genes with specific functions are shown in Supplementary Table S3.) The specific genes associated with heat treatment were classified into five groups.

### 2.4. Validation of the Results by RT-qPCR

To confirm the accuracy of the RNA-Seq transcriptome data, 10 consistently upregulated genes were selected for RT-qPCR analysis at three stages of tenderization (CON, T_1h, and T_3h). The results were consistent with the findings achieved by RNA-Seq analysis (Table 4). Single peak in the melting curve was detected, suggesting that all PCR products were specifically amplified. The change scales of the 10 gene expressions were also similar in RT-qPCR analysis when compared with those of RNA-Seq, indicating that the RNA-Seq successfully identified the DEGs.

### 2.5. Gene Ontology Analysis of DEGs

**Figure 2:**
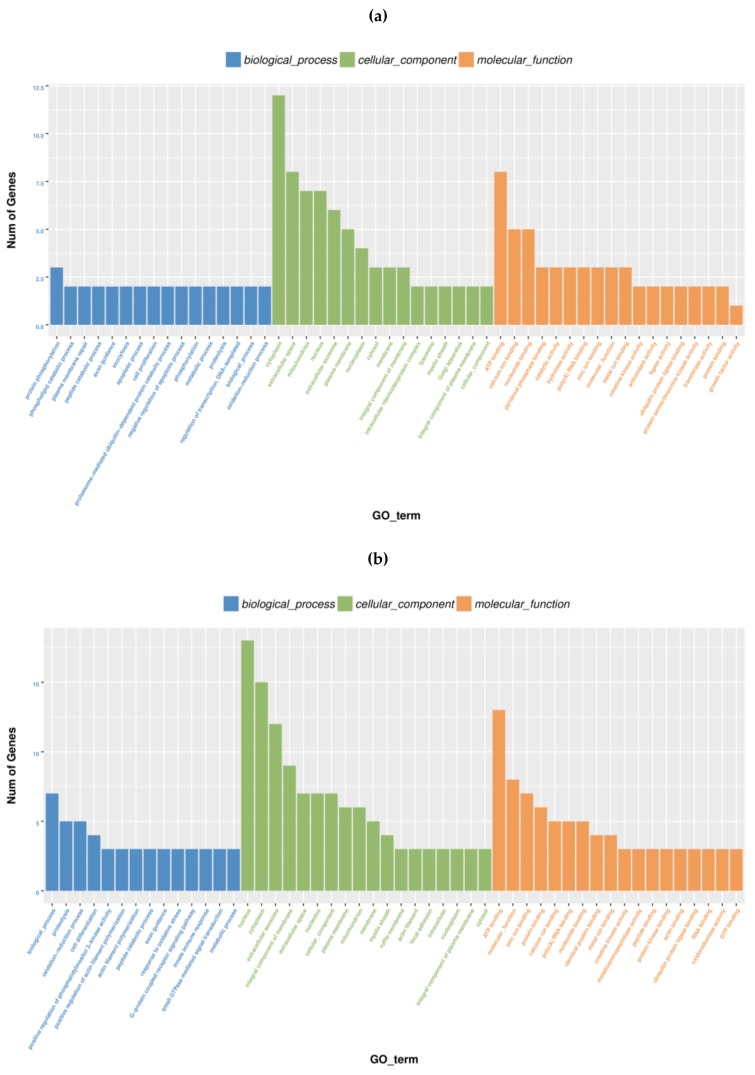
Distribution of gene ontology (GO) terms of DEGs during tenderization. The number of genes with GO-terms in the categories “Molecular function”, “Biological Process”, and “Cellular component” are shown. (**a**) GO functional classification of DEGs in T_1h vs. CON. (**b**) GO functional classification of DEGs in T_3h vs. CON.

The GO functional analysis, including “cellular component”, “molecular function”, and “biological process” involved in tenderization, was evaluated. GO terms were assigned to 328 and 487 DEGs for T_1h and T_3h, respectively (Supplementary Table S4). The top 50 terms of each GO analysis are presented in Figure 2. For T_1h vs CON, 58 DEGs could be annotated into 150 biological process, 71 cellular components, and 107 molecular functions. Similarly, between the libraries of T_3h and CON, 105 DEGs could be annotated into 247 biological process, 103 cellular components, and 137 molecular functions. With respect to the two comparisons, the terms “oxidation–reduction process” and “proteolysis” both consisted more enriched DEGs in the “biological process” category, and the terms “cytoplasm” and “extracellular space” consisted more enriched DEGs in the “cellular components” category, while the term “ATP binding” comprised the most enriched DEGs in the “molecular function” category.

### 2.6. Pathway Enrichment Analysis of DEGs

**Figure 3:**
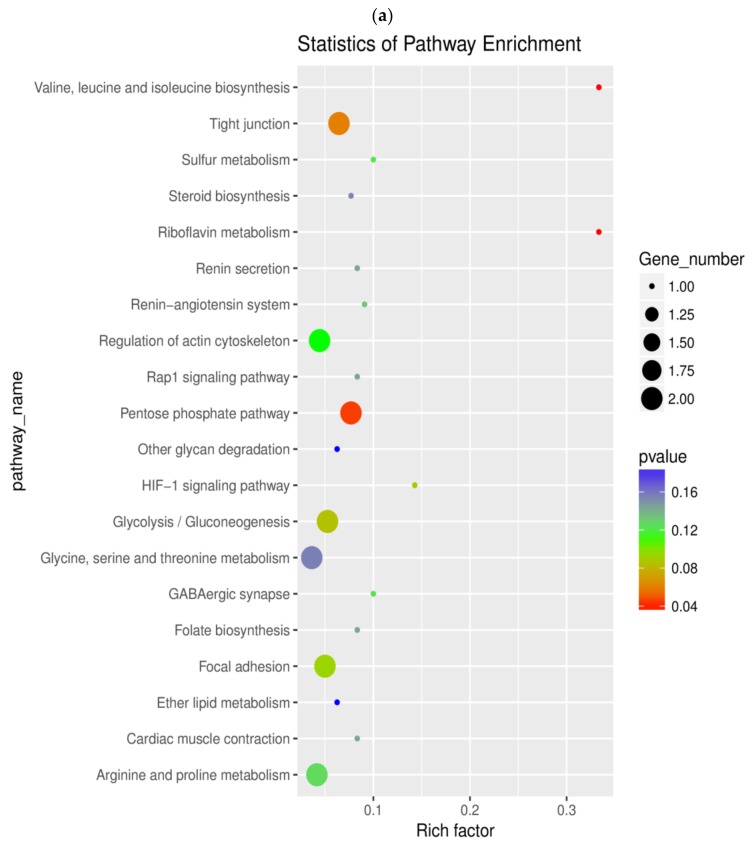

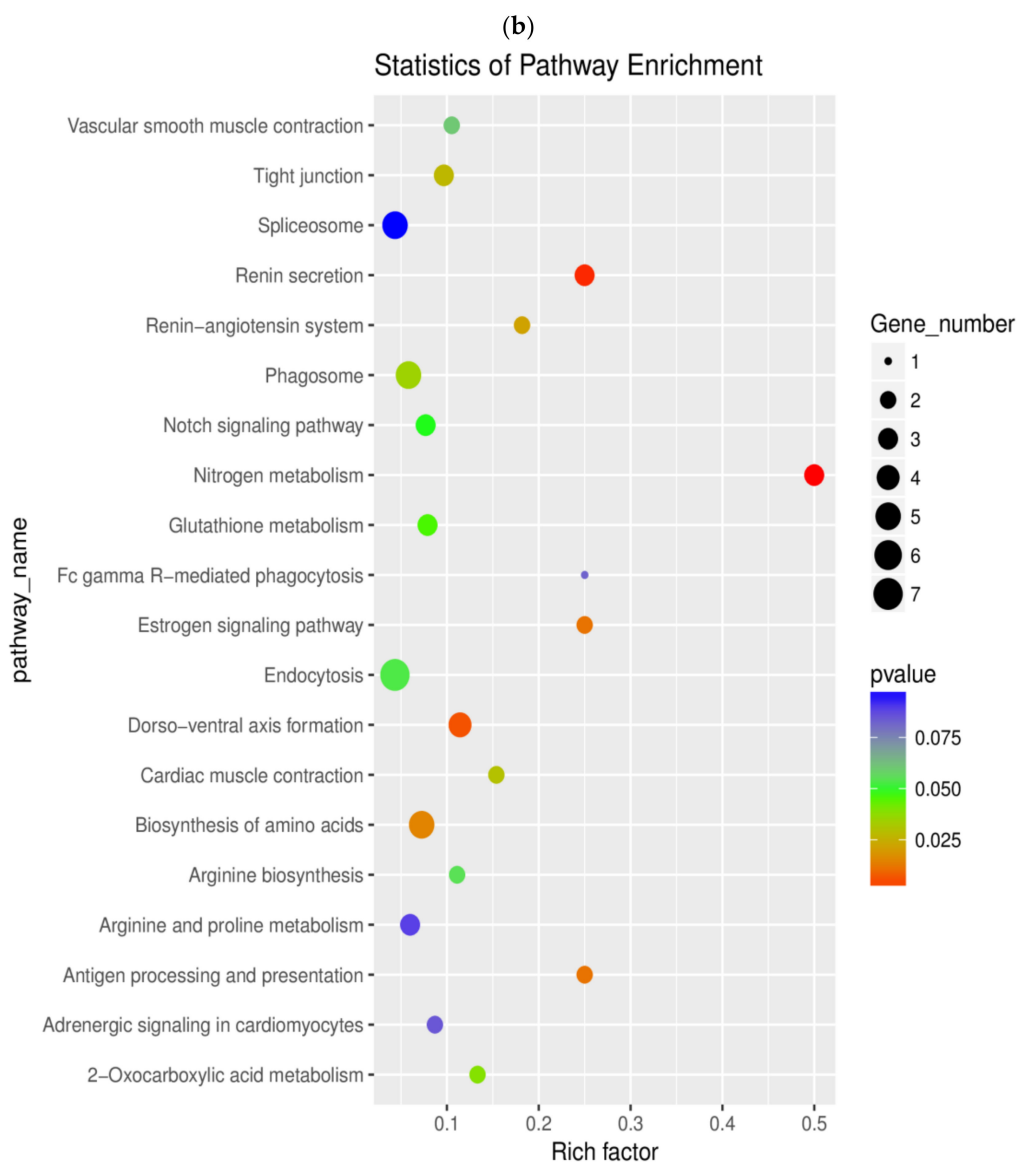
Scatterplot of the top 20 enriched Kyoto Encyclopedia of Genes and Genomes (KEGG) pathways. Rich factor represents the ratio of the number of DEGs and the number of all genes in the pathways. (**a**) KEGG pathway enrichment of DEGs in T_1h vs. CON. (**b**) KEGG pathway enrichment of DEGs in T_3h vs. CON.

To further understand the biochemical pathways involved in tenderization, enrichment analysis of DEGs was performed as demonstrated in Supplementary Table S5. The top 20 KEGG enrichment results are displayed in Figure 3. The DEGs from the two comparisons were mapped onto 54 and 69 pathways in the KEGG database, respectively, in which three and thirteen pathways were found to be enriched significantly (*p* < 0.05). In T_1h vs. CON, eleven enriched pathways were related to metabolism and three enriched pathways involving tight junction, focal adhesion, and regulation of actin cytoskeleton were related to cellular processes. Moreover, two immune-related pathways, including the hypoxia inducible factor-1 (HIF-1) signaling pathway and the Ras-proximate-1 (Rap1) signaling pathway, were also involved. In the comparison of T_3h vs CON, nine enriched pathways were related to metabolism, mainly involving nitrogen, glutathione, arginine, and proline metabolism. In addition, the most representative pathways incorporated notch the signaling pathway, antigen processing, and presentation, as well as Fc gamma R-mediated phagocytosis related to immune reaction.

## 3. Discussion

In this study, the first transcript analysis of mRNA expression levels was performed using cDNA libraries from AJBW during tenderization process using Illumina Hiseq 4000. Nearly five million valid reads sequenced in each library reached saturation as previously reported [29]. Through the analysis of the DEGs, we acquired a broad understanding of genes involved in the AJBW during tenderization. We found 631 DEGs in T_1h vs. CON and 750 DEGs in T_3h vs. CON. Thousands of variations in the transcription of genes occurred during tenderization. A number of genes related to oxidative stress response, immune response, apoptosis process, cytoskeleton, and ECM varied in terms of their expression levels during tenderization (Table 3). These results will be beneficial in future investigations of the molecular mechanism associated with tenderization in AJBW. Moreover, based on GO (functions) and KEGG (pathways) analyses, the function of DEGs during *A. japonicus* tenderization was enriched. The analyses showed that multiple GO terms and KEGG pathways were involved in proteolysis, oxidation–reduction process, metabolism, and immune reaction.

### 3.1. Genes of Endogenous Protease

In previous studies, cathepsin L and collagenase have been purified and characterized from the body wall of sea cucumber [30,31]. In addition, the gelatinolytic metalloproteinase (GMP) and cysteine protease were found to degrade the collagen protein [25,32]. In this study, we found that the genes of 72 kDa type IV collagenase and matrix metalloproteinase 16 precursor were significantly upregulated in T_1h and T_3h (Table 3). The 72 kDa type IV collagenase, also known as 72 kDa gelatinase or matrix metalloproteinase-2 (MMP-2), is an ubiquitous metalloproteinase that could degrade collagens (I, IV, V, VII, X, XI, and XIV), gelatin, elastin, fibronectin, aggrecan, and other ECM proteins [27]. Matrix metalloproteinase-16 (MMP-16), also called MT3-MMP, can degrade various components of the ECM, such as collagen type III, gelatin, casein, and fibronectin. The AJBW dermis is a typical catch connective tissue (or called mutable collagenous tissue) that contains a large amount of ECM consisting mainly of collagen fibrils, proteoglycans and microfibrils [33]. Approximately 70% of the total body wall protein was insoluble collagen fibers and the collagen protein belonged to collagen type I formed by (α1)_2_α2 [34]. The noncollagenous protein from the body wall of sea cucumber contains glycoprotein (400 kDa) as the main component exhibiting structural similarity to fibronectins from other vertebrate and invertebrate animals [35]. With respect to the results obtained, we inferred that MMP-2 and MMP-16 were the main endogenous proteases that facilitated the tenderization process by degrading the ECM of the AJBW. Moreover, the processes of oxidative stress, immune response, apoptosis and reorganization of cytoskeleton and ECM were involved during tenderization in AJBW, which might be attributed to activation of MMPs. This is inconsistent with previous studies that also concluded that MMP gene expression may be modulated in both physiological and pathophysiological events [36].

### 3.2. Genes Associated with Oxidative Stress Response

Numerous studies on marine animals, such as coral [37], pacific oyster [38], and marine snail [39], have reported that exposure to thermal stress can induce reactive oxygen species (ROS) accumulation following antioxidant defense mechanism. In our study, oxidant response genes, such as glutathione S-transferase (GST) and glutathione peroxidase (GPx), showed significant upregulated patterns (Table 3), and superoxide dismutase, catalase, and microsomal GST decreased in AJBW during the heating period of 3 h. Similarly, we previously detected two times increment in signal intensity of ROS-derived radicals in T_1h vs CON [40]. Previous studies reported that the transcripts and activities of the antioxidant enzymes could be altered differently by temperature gradients combined with exposure periods [41,42,43]. Moreover, oxidative stress could induce apoptosis [44], autophagy [45], and the immune response [46] by stimulating various types of cascade reactions, besides regulating collagen synthesis and MMPs activities [47]. Our results indicated that the response to oxidative stress induced by heat stress was essential in AJBW during tenderization.

### 3.3. Genes Associated with Immune Response

Innate immune system is the primary immune system for invertebrates due to the lack of acquired or adaptive immune system to defense against the pathogens [48]. As mentioned before, MMPs could be regulated by some cytokines (IL-1β) and transforming growth factor-β1 (TGF-β1) [27], in addition to some specific MMPs that can control chemokine activity [49]. This suggested that immune response could have been stimulated in AJBW during tenderization.

The superfamily of fibrinogen-related proteins (FREPs), such as fibrinogen-like protein, tenascin, ficolin, angiopoietin, and hepassocin, have been reported to play vital roles in innate immune responses [50] and regeneration [51] in invertebrates. Previous studies have also demonstrated that FREP A is widely distributed in the body wall, intestines, longitudinal muscles and respiratory channel of *A. japonicus* [52]. In our study, we found that the genes of FREP A and fibrinogen C domain-containing protein 1 were upregulated, and the genes of ficolin-2 and tenascin-N were downregulated in AJBW during tenderization. FREPs are supposed to exhibit ‘antibody-like’ properties in biological mechanisms of recognition and binding of invading pathogens in marine animals like shrimps [53], mussels [54], and marine snails [55]. The roles of FREPs in AJBW during tenderization remain unclear and further investigations are required to reveal their function.

In invertebrates, the complement system is an innate immune response that attacks the surfaces of foreign cells [56]. It consists of three complement pathways such as classical, alternative and lectin pathways. C3 genes have been involved in the three pathways, and Bf genes, that can activate C3, have been involved in the alternative pathway [57]. In our results, the genes of C3, Bf, and Bf-2 were upregulated in T_1h vs. CON and T_3h vs CON. Some previous studies also reported C3-2 could be triggered in *A. japonicus* by LPS challenge [58] or *Vibrio splendidus* [59]. However, we could not identify this gene in our transcript libraries. Similarly, an important component of the lectin complement pathway—mannose-binding lectin (MBL)—was also not identified. However, lactose-binding lectins (LBL) and mannan-binding lectin serine protease (MASP) were found to be upregulated in tenderized AJBW (Table 3). It is not known that whether the LBL (just like MBL or ficolin) would combine with MASP to activate C3, thus, further investigation is required. Although it is possible that these complement pathways could be stimulated in AJBW during tenderization that would trigger the terminal pathway to generate membrane attack complex (MAC) affecting the permeabilities of biofilms. Besides, it has been demonstrated that MAC can both induce apoptosis leading to tissue damage [60] and clear of apoptotic cells [56], which suggested that the complement system may be related to apoptosis in AJBW during tenderization.

In *A. japonicus*, heat shock proteins (HSPs) as a bioindicator of thermal stress, could act as innate immune agents and stimulate the immune system [61]. They are produced by the cells in response to environmental stress factors such as heat shock [62], osmotic stress [63], and ultraviolet light [64]. In this study, four encode HSP70 mRNA were significantly upregulated during the tenderization process. This is in consistence with other reports on *A. japonicus* [65] and chocolate chip cucumber, *Isostichopus badionotus* [43], in which respective genes were upregulated due to heat stress. HSP70 had been found to be related to apoptosis [66] and protein degradation [67]. Thus, it can be concluded that the thermal treatment could enhance the expression of HSP70 and was critical to *A. japonicus* during tenderization.

### 3.4. Genes Associated with Apoptosis Process

Cell apoptosis could be induced by thermal treatment in marine animals [37]. Similarly, MMPs could also affect apoptosis [68]. In our study, based on the GO annotation, two genes encoding apoptosis-related proteins (poly-U-binding factor 60 kDa (PUF60) and TFIIH basal transcription factor complex helicase XPB) were detected and upregulated during tenderization. The first echinoderm PUF60 was identified in gonad, coelomocytes, intestine, respiratory tree, and body wall of sea cucumber *Stichopus monotuberculatus*. Moreover, the overexpressed PUF60 could induce apoptosis [69]. In addition, two antiapoptosis-related genes (Bax inhibitor-1 and Src family kinase 5) were also identified with downregulated expression during tenderization. Bax belongs to the Bcl-2 family that governs the mitochondrial outer membrane permeabilization and performs proapoptotic function. Bax inhibitor-1 is located in endoplasmic reticulum (ER) membranes and protects the cells from ER stress-induced apoptosis [70]. Src family kinase (belonging to family of non-receptor tyrosine kinases) could inhibit cytokines activation and control the cell apoptosis process [71]. The downregulation of these two genes (Bax inhibitor-1 and Src family kinase 5) further indicated that the original antiapoptosis mechanisms were damaged and irreversible apoptosis occurred in the cells of tenderized AJBW. These results were supported by our previous findings about the DNA damage and enhancement of caspase-3 activity in AJBW during tenderization [40].

### 3.5. Genes Associated with Reorganization of Cytoskeleton and ECM

Thermal treatment can affect the ultrastructure of the cell, which has been found in catfish [72], scallops [73], and corals [74]. Genes encoding cytoskeleton-associated proteins, such as myosin, actin, titin, troponin, tropomodulin, and gelsolin, were upregulated during tenderization (Table 3). Cell volume could be adjusted through manipulation of transporters and cytoskeletal reorganization, which may affect the phagocytic activity [58]. Three genes encoding myosin light chain kinase smooth muscle were upregulated during tenderization, which could have phosphorylated the myosin light chain causing the contractile activity of the smooth muscle [75]. In addition, it has been proved that actin can protect the cells from oxidative stress [76], indicating that the upregulation of actin may build the defense against oxidative damage. The exposure to thermal treatment also induced expression of genes involved in ECM synthesis and remodeling including α collagen gene and MMP-2, a MMP-16 precursor. Besides, integrin β (an important transmembrane receptor in immune systems) was also upregulated (Table 3). The integrin signaling can regulate ECM remodeling and control subsequent cell behavior and tissue organization [77]. Thus, it can be suggested that ECM reorganization was induced in AJBW during tenderization.

## 4. Materials and Methods

### 4.1. Animals Materials

Live *A. japonicus* (150 ± 20 g, body weight) were procured from a local aquatic market in Dalian, China. The viscera and heads were removed and each body wall was cut into large pieces and transferred onto the petri plates with three pieces per plate before incubating at 37 °C for 1 h (T_1h) and 3 h (T_3h). This temperature is suitable to activate endogenous enzymes activities. All the body wall chunks, including the incubated and fresh as control samples (CON), were frozen immediately with liquid nitrogen and then stored at −80 °C until RNA extraction. 

### 4.2. mRNA Library Construction and Sequencing

Total RNA was extracted from nine samples (three biological replicates each of CON, T_1h and T_3h) using miRNeasy minikit (Qiagen, Duesseldorf, Germany) following the manufacturer’s instructions. The quantity and purity of the total RNA were determined using Bioanalyzer 2100 and RNA 6000 Nano Lab Chip Kit (Agilent, Santa Clara, CA, USA) with RIN > 7. Approximately 10 μg of total RNA was used for transcriptome cDNA library construction using mRNA Seq sample preparation kit (Illumina, San Diego, CA, USA). The average insert size for the paired-end libraries was 300 bp (±50 bp). Then we performed the paired-end sequencing on an Illumina Hiseq 4000 (LC Sciences, Houston, TX, USA) following the manufacturer’s recommended protocol and generated a total of million paired-end reads of 150 bp length. Prior to assembly, the low quality reads (reads containing sequencing adaptors; reads containing sequencing primer; and nucleotides with *q* quality score < 20) were removed. The raw sequence data have been submitted to the NCBI Short Read Archive [78] under accession code GSE123077.

### 4.3. RNA-seq Reads Mapping and DEG Testing

We aligned the reads of CON, T_1h and T_3h with *A. japonicus* reference genome [28] using HISAT package that initially removes a portion of the reads based on quality information accompanying each read and then maps the reads to the reference genome. The mapped reads were assembled using StringTie and merged to reconstruct a comprehensive transcriptome using perl scripts. Later, the expression levels of all transcripts were estimated using StringTie and Ballgown gene expression levels were calculated according to FPKM (Fragments Per Kilobase of exon model per Million mapped reads). The DEGs were selected with log2 (fold change) >1 or log2 (fold change) < −1 with statistical significance (*p* < 0.05) using R package—Ballgown. The genes were searched using Nr, SwissProt protein database, and Pfam database with E value ≤ 10^−5^. To further identify the functional classifications, all the genes were mapped to the GO database [79] and KEGG resource [80] with a corrected *p*-value ≤ 0.05 and a *Q* ≤ 0.05, respectively.

### 4.4. RT-qPCR Validation

To validate the RNA-seq results, 10 DEGs were employed on the qPCR software (version 3.0, qTOWER 2.2, Janalytik Jena, Jena, Germany). Total RNA was isolated from three individuals in each stage (CON, T_1h and T_3h) and was reverse-transcribed into cDNA templates using the PrimeScript™ RT reagent Kit with gDNA Eraser (TaKaRa, Otsu, Japan) according to the manufacturer’s instructions. The reaction condition consists of two steps: 37 °C for 15 min and then 85 °C for 5 s. The primer pairs for the selected DEGs are listed in Supplementary Table S6. Amplification of cytochrome b (Cyt b) gene was selected as the reference gene [61]. Each sample run was carried out in triplicate, along with Cyt b. The RT-qPCR amplification was performed in a volume of 20 μL containing 10 μL of 2× TB Green Premix Ex Taq™ II, 2 μL of cDNA template, 1 μL each of forward and reverse primers (10 μM), and 6 μL deionized water using TB Green^TM^ Premix Ex Taq™ II Kit (Tli RNase H Plus, TaKaRa, Otsu, Japan) according to the manufacturer’s instructions with slight modification. The PCR conditions were 95 °C for 30 s, 40 cycles (heating at 95 °C for 5 s and annealing at 60 °C for 30 s), 95 °C for 30 s, 60 °C for 30 s, and 95 °C for 15 s. The 2^−ΔΔCT^ method was used to analyze the comparative expression levels. Statistical analysis was performed using SPSS 16.0 software (IBM Corp., Armonk, NY, USA) with the test conducted as a two-tailed Independent Samples *t*-test and a significance level of *p* < 0.05.

## 5. Conclusions

In this study, we used RNA-seq to identify the various DEGs in the tenderized AJBW and found that the genes of MMP-2 and MMP-16 were significantly upregulated which could efficiently explain the changes in tenderness in AJBW during 37 °C treatment. Moreover, the results suggested that the tenderization is a complex process that involved oxidative stress, immune response, autolysis, and reorganization of the cytoskeleton and ECM. These specific relationships of the regulatory mechanisms and endogenous protease activation warrant further investigations that can help to further improve the thermal processes required to maintain the high quality of *A. japonicus*.

## Figures and Tables

**Table 1:** Characteristics of the reads from nine different grade follicle libraries in *A. japonicus* body wall (AJBW).

Sample	Raw Data		Clean Data		Valid Ratio %	Q20 %	Q30 %	GC Content %
	Read	Base	Read	Base	Read			
CON_1	53397180	8.01G	52327150	7.85G	98.00	99.40	93.73	42
CON_2	54956050	8.24G	53785462	8.07G	97.87	99.57	94.67	41
CON_3	58609160	8.79G	57471652	8.62G	98.06	99.25	92.29	40
T_1h_1	48381506	7.26G	47514898	7.13G	98.21	99.57	94.62	43
T_1h_2	56821096	8.52G	55435722	8.32G	97.56	99.02	91.34	41
T_1h_3	55081090	8.26G	53965518	8.09G	97.97	99.38	93.39	42
T_3h_1	50974702	7.65G	50010072	7.50G	98.11	99.20	92.92	41
T_3h_2	56378238	8.46G	54928996	8.24G	97.43	99.42	94.27	42
T_3h_3	51550962	7.73G	50534502	7.58G	98.03	99.38	92.95	43

Q20, base sequencing error probability <1%; Q30, base sequencing error probability <0.1%.

**Table 2:** Summary of RNA-seq alignment.

Sample	Valid Reads	Mapped Reads	Unique Mapped Reads	Multimapped Reads
CON_1	52327150	40400777(77.21%)	22317749(42.65%)	18083028(34.56%)
CON_2	53785462	40377886(75.07%)	23759345(44.17%)	16618541(30.90%)
CON_3	57471652	41429455(72.09%)	24918806(43.36%)	16510649(28.73%)
T_1h_1	47514898	33468336(70.44%)	18785041(39.54%)	14683295(30.90%)
T_1h_2	55435722	41648386(75.13%)	23013370(41.51%)	18635016(33.62%)
T_1h_3	53965518	40764443(75.54%)	24024127(44.52%)	16740316(31.02%)
T_3h_1	50010072	38674656(77.33%)	16814094(33.62%)	21860562(43.71%)
T_3h_2	54928996	45056381(82.03%)	22811470(41.53%)	22244911(40.50%)
T_3h_3	50534502	36913545(73.05%)	22295211(44.12%)	14618334(28.93%)

**Table 3:** Special types of the main DEGs in AJBW during tenderization.

Gene ID	Gene Name	T_1h vs. CON		T_3h vs. CON	
		**log2(fc)**	***p***	**log2 (fc)**	***p***
*Endogenous Protease*					
MSTRG.28936	72 kDa type IV collagenase	**1.92**	**0.03**	**2.22**	**0.03**
MSTRG.5390	MMP 16 precursor	**1.57**	**0.04**	**1.50**	**0.03**
*Oxidative Stress Response*					
MSTRG.45929	Glutathione S-transferase theta-1-like	**1.20**	**0.04**	**1.68**	**0.01**
MSTRG.38457	Microsomal glutathione S-transferase 1	−0.35	0.18	**−0.99**	**0.02**
MSTRG.1616	Glutathione peroxidase	**1.08**	**0.02**	**1.75**	**0.02**
*Immune System Process*					
MSTRG.9314	Fibrinogen-like protein A	**2.00**	**0.00**	**1.57**	**0.04**
MSTRG.14673	Fibrinogen C domain-containing protein 1-like	0.76	0.29	**1.04**	**0.03**
MSTRG.30809	Ficolin-2	−1.51	0.29	**−2.49**	**0.03**
MSTRG.40130	Tenascin-N	**−1.93**	**0.05**	0.93	0.63
MSTRG.13644	Complement component C3	**3.29**	**0.02**	**3.19**	**0.01**
MSTRG.2787	Complement factor B	**2.49**	**0.00**	**2.24**	**0.03**
MSTRG.3468	Complement factor B-2	1.81	0.17	**2.02**	**0.04**
MSTRG.16733	Mannan-binding lectin serine protease 1-like	**1.39**	**0.00**	**1.45**	**0.03**
MSTRG.25958	Lactose-binding lectin l-2-like	**1.58**	**0.03**	1.21	0.16
MSTRG.37300	HSP70	**1.14**	**0.04**	**1.87**	**0.00**
MSTRG.6868	Integrin beta-like protein A	**3.63**	**0.00**	**3.47**	**0.00**
MSTRG.21840	Integrin alpha-4	−0.69	0.14	**−1.50**	**0.05**
*Apoptosis*					
MSTRG.20169	PUF60 isoform X3	**1.20**	**0.04**	1.04	0.19
MSTRG.4067	TFIIH basal transcription factor complex helicase XPB subunit-like	0.67	0.09	**1.33**	**0.02**
MSTRG.14930	Bax inhibitor-1	**−0.65**	**0.02**	**−1.30**	**0.03**
MSTRG.9235	Src family kinase 5	−0.72	0.05	**−1.14**	**0.04**
*Cytoskeleton and ECM associated genes*					
MSTRG.2582	Myosin heavy chain, striated muscle isoform X5	**1.72**	**0.03**	**2.14**	**0.00**
MSTRG.23618	Myosin heavy chain, striated muscle isoform X7	**2.25**	**0.01**	**2.39**	**0.01**
MSTRG.21690	Myosin-10	**1.07**	**0.01**	**0.61**	**0.00**
MSTRG.13638	Unconventional myosin-X	0.65	0.11	1.16	0.03
MSTRG.36459	Myosin-2 essential light chain-like	1.34	0.15	**2.33**	**0.01**
MSTRG.2896	Myosin light chain kinase, smooth muscle	2.03	0.06	**2.35**	**0.01**
MSTRG.23243	Actin isoform 2	1.34	0.11	**1.65**	**0.01**
MSTRG.7111	Actin	−0.40	0.55	**−1.14**	**0.00**
MSTRG.30094	Titin	**2.10**	**0.02**	**3.00**	**0.01**
MSTRG.18253	Troponin I, partial	**1.64**	**0.03**	**1.93**	**0.01**
MSTRG.29309	Tropomodulin-1-like	−0.57	0.13	**−1.10**	**0.04**
MSTRG.30449	Gelsolin-like protein 2	1.23	0.17	**2.04**	**0.00**
MSTRG.37982	Alpha-1 collagen isoform X4	**1.27**	**0.04**	**1.63**	**0.04**
MSTRG.5905	Alpha-2 collagen	**1.75**	**0.03**	**2.48**	**0.01**
MSTRG.28962	Collagen alpha-1(V) chain	**1.33**	**0.00**	1.29	0.15
MSTRG.41401	Collagen IV alpha-3-binding protein-like	**1.38**	**0.04**	1.22	0.21

The significant data is bold (*p* < 0.05). FC, fold change.

**Table 4:** Expression patterns of the 10 mRNAs selected for RT-qPCR validation.

Gene ID	T_1h vs. CON		T_3h vs. CON	
	RNA-Seq	RT-qPCR	RNA-Seq	RT-qPCR
MSTRG.6868	3.63 **	1.75 **	3.47 **	2.69 **
MSTRG.13644	3.29 *	2.95 **	3.19 *	2.03 **
MSTRG.23313	1.97 **	2.26 *	2.04 *	5.55 **
MSTRG.32095	1.94 **	3.05 **	1.77 **	3.51 *
MSTRG.28936	1.92 *	1.55 *	2.22 *	1.26 *
MSTRG.22626	1.84 *	2.15 *	1.87 *	3.47 **
MSTRG.18184	1.67 **	2.14 **	1.38 *	4.71 **
MSTRG.5390	1.57 *	1.23 *	1.50 *	2.01 *
MSTRG.16733	1.39 **	1.67 *	1.45 *	2.31 **
MSTRG.37982	1.27 *	2.00 *	1.63 *	9.68 **

* *p* < 0.05; ** *p* < 0.01.

## Supplementary Materials

Supplementary materials can be found available online.

## Abbreviations

AJBW	*Apostichopus japonicus* body wall
Cyt b	Cytochrome b
DEGs	differential expression genes
ECM	extracellular matrix
ER	endoplasmic reticulum
FPKMs	Fragments Per Kilobase of exon model per Million mapped reads
FREPs	fibrinogen-related proteins
Gb	giga bases
GMP	gelatinolytic metalloproteinase
GO	Gene Ontology
GPx	glutathione peroxidase
GST	glutathione S-transferase
HIF-1	hypoxia inducible factor-1
HISAT	Hierarchical Indexing for Spliced Alignment of Transcripts
HSPs	heat shock proteins
IL-1	interleukin-1
KEGG	Kyoto Encyclopedia of Genes and Genomes
LBLLPS	lactose-binding lectinslipopolysaccharide
MAC	membrane attack complex
MASP	mannan-binding lectin serine protease
MBL	mannose-binding lectin
MMP	matrix metalloproteinase
Nr	nonredundant protein sequences
PUF60	poly-U-binding factor 60 kDa
RT-qPCR	quantitative Real-time Polymerase Chain Reaction
Rap-1	Ras-proximate-1
ROS	reactive oxygen species
TGF-β1	transforming growth factor-1
TNF	tumor necrosis factor

## References

[1] Wu H.T., Li D.M., Zhu B.W., Sun J.J., Zheng J., Wang F.L., Konno K., Jiang X. (2013). Proteolysis of noncollagenous proteins in sea cucumber, *Stichopus japonicus*, body wall: Characterisation and the effects of cysteine protease inhibitors. Food Chem..

[2] Bureau of Fisheries in Ministry of Agriculture (2017). China Fishery Statistical Yearbook.

[3] Qi H., Fu H., Dong X., Feng D., Li N., Wen C., Nakamura Y., Zhu B. (2016). Apoptosis induction is involved in UVA-induced autolysis in sea cucumber *Stichopus japonicus*. J. Photochem. Photobiol. B.

[4] Qi H., Dong X.F., Zhao Y.P., Li N., Fu H., Feng D.D., Liu L., Yu C.X. (2016). ROS production in homogenate from the body wall of sea cucumber *Stichopus japonicus* under UVA irradiation: ESR spin-trapping study. Food Chem..

[5] Christensen L., Gunvig A., Torngren M.A., Aaslyng M.D., Knochel S., Christensen M. (2012). Sensory characteristics of meat cooked for prolonged times at low temperature. Meat Sci..

[6] Huang M., Huang F., Xue M., Xu X., Zhou G. (2011). The effect of active caspase-3 on degradation of chicken myofibrillar proteins and structure of myofibrils. Food Chem..

[7] Dong X.P., Li Y., Li Y., Song L., Cheng S.S., Li D.M., Zhu B.W., Zhou D.Y., Tan M.Q. (2016). Combination of NMR and MRI techniques for non-invasive assessment of sea cucumber (*Stichopus japonicas*) tenderization during low-temperature heating process. Food Anal. Methods.

[8] Bi J.R., Li Y., Cheng S.S., Dong X.P., Kamal T., Zhou D.Y., Li D.M., Jiang P.F., Zhu B.W., Tan M.Q. (2016). Changes in body wall of sea cucumber (*Stichopus japonicus*) during a two-step heating process assessed by rheology, LF-NMR, and texture profile analysis. Food Biophys..

[9] Zhong C., Cai Q.F., Liu G.M., Sun L.C., Hara K., SU W.J., Cao M.J. (2012). Purification and characterisation of cathepsin L from the skeletal muscle of blue scad (*Decapterus maruadsi*) and comparison of its role with myofibril-bound serine proteinase in the degradation of myofibrillar proteins. Food Chem..

[10] Wang K.K. (2000). Calpain and caspase: Can you tell the difference?. Trends Neurosci..

[11] Chen L., Feng X.C., Zhang Y.Y., Liu X.B., Zhang W.G., Li C.B., Ullah N., Xu X.L., Zhou G.H. (2015). Effects of ultrasonic processing on caspase-3, calpain expression and myofibrillar structure of chicken during post-mortem ageing. Food Chem..

[12] Dong X.P., Zhu B.W., Sun L.M., Zheng J., Jiang D., Zhou D.Y., Wu H.T., Murata Y. (2011). Changes of collagen in sea cucumber (*Stichopus japonicas*) during cooking. Food Sci. Biotechnol..

[13] Qin L., Bi J.R., Li D.M., Dong M., Zhao Z.Y., Dong X.P., Zhou D.Y., Zhu B.W. (2016). Unfolding/refolding study on collagen from sea cucumber based on 2D fourier transform infrared spectroscopy. Molecules.

[14] Huang F., Huang M., Zhang H., Zhang C., Zhang D., Zhou G. (2016). Changes in apoptotic factors and caspase activation pathways during the postmortem aging of beef muscle. Food Chem..

[15] Wang L.L., Han L., Ma X.L., Yu Q.L., Zhao S.N. (2017). Effect of mitochondrial apoptotic activation through the mitochondrial membrane permeability transition pore on yak meat tenderness during postmortem aging. Food Chem..

[16] Tan M.H., Au K.F., Yablonovitch A.L., Wills A.E., Chuang J., Baker J.C., Wong W.H., Li J.B. (2013). RNA sequencing reveals a diverse and dynamic repertoire of the *Xenopus tropicalis* transcriptome over development. Genome Res..

[17] Sun L., Chen M., Yang H., Wang T., Liu B., Shu C., Gardiner D.M. (2011). Large scale gene expression profiling during intestine and body wall regeneration in the sea cucumber *Apostichopus japonicus*. Comp. Biochem. Physiol. Part D Genom. Proteom..

[18] Li C., Feng W., Qiu L., Xia C., Su X., Jin C., Zhou T., Zeng Y., Li T. (2012). Characterization of skin ulceration syndrome associated microRNAs in sea cucumber *Apostichopus japonicus* by deep sequencing. Fish Shellfish Immunol..

[19] Zhao Y., Yang H., Storey K.B., Chen M. (2014). Differential gene expression in the respiratory tree of the sea cucumber *Apostichopus japonicus* during aestivation. Mar. Genom..

[20] Zhou Z.C., Dong Y., Sun H.J., Yang A.F., Chen Z., Gao S., Jiang J.W., Guan X.Y., Jiang B., Wang B. (2014). Transcriptome sequencing of sea cucumber (*Apostichopus japonicus*) and the identification of gene-associated markers. Mol. Ecol. Resour..

[21] Li Y., Huang J., Liu Z., Zhou Y., Xia B., Wang Y., Kang Y., Wang J. (2017). Transcriptome analysis provides insights into hepatic responses to moderate heat stress in the rainbow trout (*Oncorhynchus mykiss*). Gene.

[22] Shiel B.P., Hall N.E., Cooke I.R., Robinson N.A., Strugnell J.M. (2015). De novo characterisation of the greenlip abalone transcriptome (*Haliotis laevigata*) with a focus on the heat shock protein 70 (HSP70) family. Mar. Biotechnol..

[23] Barshis D.J., Ladner J.T., Oliver T.A., Palumbi S.R. (2014). Lineage-specific transcriptional profiles of *Symbiodinium* spp. unaltered by heat stress in a coral host. Mol. Biol. Evol..

[24] Smolina I., Kollias S., Møller E.F., Lindeque P., Sundaram A.Y.M., Ferandes J.M.O., Hoarau G. (2015). Contrasting transcriptome response to thermal stress in two key zooplankton species, *Calanus finmarchicus* and *C. glacialis*. Mar. Ecol. Prog. Ser..

[25] Liu Y.X., Zhou D.Y., Ma D.D., Liu Z.Q., Liu Y.F., Song L., Dong X.P., Li D.M., Zhu B.W., Konno K. (2017). Effects of endogenous cysteine proteinases on structures of collagen fibres from dermis of sea cucumber (*Stichopus japonicus*). Food Chem..

[26] Sun L.M., Wang T.T., Zhu B.W., Niu H.L., Zhang R., Hou H.M., Zhang G.L., Murata Y. (2013). Effect of matrix metalloproteinase on autolysis of sea cucumber *Stichopus japonicus*. Food Sci. Biotechnol..

[27] Zitka O., Kukacka J., Krizkova S., Huska D., Adam V., Masarik M., Prusa R., Kizek R. (2010). Matrix metalloproteinases. Curr. Med. Chem..

[28] The Sea Cucumber *Apostichopus japonicus* Genome. http://www.genedatabase.cn/aja_genome_20161129.html.

[29] Zhang L.B., Feng Q.M., Sun L.N., Fang Y., Xu D.X., Zhang T., Yang H.S. (2018). Differential gene expression in the body wall of the sea cucumber (*Apostichopus japonicus*) under strong lighting and dark conditions. Acta Oceanol. Sin..

[30] Zhu B.W., Zhao L.L., Sun L.M., Li D.M., Murata Y., Yu L., Zhang L. (2014). Purification and characterization of a cathepsin L-like enzyme from the body wall of the sea cucumber *Stichopus japonicus*. Biosci. Biotechnol. Biochem..

[31] Zhong M., Hu C., Ren C., Luo X., Cai Y. (2015). Characterization of a main extracellular matrix autoenzyme from the dermis of sea cucumber *Stichopus monotuberculatus*: Collagenase. Int. J. Food Prop..

[32] Wu H.L., Hu Y.Q., Shen J.D., Cai Q.F., Liu G.M., Su W.J., Cao M.J. (2013). Identification of a novel gelatinolytic metalloproteinase (GMP) in the body wall of sea cucumber (*Stichopus japonicus*) and its involvement in collagen degradation. Process Biochem..

[33] Szlugit G. (2007). The echinoderm collagen fibril: A hero in the connective tissue research of the 1990s. Bioessays.

[34] Saito M., Kunisaki N., Urano N., Kimura S. (2002). Collagen as the major edible component of sea cucumber (*Stichopus japonicus*). J. Food Sci..

[35] Mizuta S., Koizumi Y., Inoue S., Someya C., Hosoi M., Yokoyama Y., Yoshinaka R. (2013). Existence of a 400kDa glycoprotein in the dermis of sea cucumber *Apostichopus armata*: Partial purification and characterization. Fish Sci..

[36] Tallant C., Nodarse A.M., Gomis-Rüth F.X. (2009). Matrix metalloproteinases: Fold and function of their catalytic domains. Biochim. Biophys. Acta Mol. Cell Res..

[37] Barshis D.J., Ladner J.T., Oliver T.A., Seneca F.O., Traylor-Knowles N., Palumbi S.R. (2013). Genomic basis for coral resilience to climate change. Proc. Natl. Acad. Sci. USA.

[38] Lim H.J., Kim B.M., Hwang I.J., Lee J.S., Choi I.Y., Kim Y.J., Rhee J.S. (2016). Thermal stress induces a distinct transcriptome profile in the Pacific oyster *Crassostrea gigas*. Comp. Biochem. Physiol. Part D Genom. Proteom..

[39] Gleason L.U., Burton R.S. (2015). RNA-seq reveals regional differences in transcriptome response to heat stress in the marine snail *Chlorostoma funebralis*. Mol. Ecol..

[40] Dong X.F., Qi H., Feng D.D., He B.Y., Nakamura Y., Yu C.X., Zhu B.W. (2018). Oxidative stress involved in textural changes of sea cucumber *Stichopus japonicus* body wall (SJBW) during low-temperature treatment. Int. J. Food Prop..

[41] Lang R.P., Bayne C.J., Camara M.D., Cunningham C., Jenny M.J., Langdon C.J. (2009). Transcriptome profiling of selectively bred Pacific oyster *Crassostrea gigas* families that differ in tolerance of heat shock. Mar. Biotechnol..

[42] Meistertzheim A.L., Tanguy A., Moraga D., Thebault M.T. (2007). Identification of differentially expressed genes of the Pacific oyster *Crassostrea gigas* exposed to prolonged thermal stress. FEBS J..

[43] Gullian Klanian M., Terrats Preciat M. (2017). Effect of pH on temperature controlled degradation of reactive oxygen species, heat shock protein expression, and mucosal immunity in the sea cucumber *Isostichopus badionotus*. PLoS ONE.

[44] Mates J.M. (2000). Effects of antioxidant enzymes in the molecular control of reactive oxygen species toxicology. Toxicol.

[45] Chen Y., Azad M.B., Gibson S.B. (2009). Superoxide is the major reactive oxygen species regulating autophagy. Cell Death Differ..

[46] Habte-Tsion H.M., Ren M., Liu B., Ge X., Xie J., Chen R. (2016). Threonine modulates immune response, antioxidant status and gene expressions of antioxidant enzymes and antioxidant-immune-cytokine-related signaling molecules in juvenile blunt snout bream (*Megalobrama amblycephala*). Fish Shellfish Immunol..

[47] Siwik D.A., Pagano P.J., Colucci W.S. (2001). Oxidative stress regulates collagen synthesis and matrix metalloproteinase activity in cardiac fibroblasts. Am. J. Physiol. Cell Physiol..

[48] Hoffmann J.A., Kafatos F.C., Janeway C.A., Ezekowitz R.A. (1999). Phylogenetic perspectives in innate immunity. Science.

[49] Parks W.C., Wilson C.L., Lopez-Boado Y.S. (2004). Matrix metalloproteinases as modulators of inflammation and innate immunity. Nat. Rev. Immunol..

[50] Ramirez-Gomez F., Ortiz-Pineda P.A., Rivera-Cardona G., Garcia-Arraras J.E. (2009). LPS-induced genes in intestinal tissue of the sea cucumber *Holothuria glaberrima*. PLoS ONE.

[51] Zhang X., Sun L., Yuan J., Sun Y., Gao Y., Zhang L., Li S., Dai H., Hamel J.F., Liu C. (2017). The sea cucumber genome provides insights into morphological evolution and visceral regeneration. PLoS Biol..

[52] Wu Y., Yao F., Mei Y., Chu B., Cheng C., Liu Y., Li X., Zou X., Hou L. (2014). Cloning and expression analysis of the gene encoding fibrinogen-like protein A, a novel regeneration-related protein from *Apostichopus japonicus*. Mol. Biol. Rep..

[53] Soonthornchai W., Chaiyapechara S., Klinbunga S., Thongda W., Tangphatsornruang S., Yoocha T., Jarayabhand P., Jiravanichpaisal P. (2016). Differentially expressed transcripts in stomach of *Penaeus monodon* in response to AHPND infection. Dev. Comp. Immunol..

[54] Tanguy M., Gauthier-Clerc S., Pellerin J., Danger J.M., Siah A. (2018). The immune response of *Mytilus edulis* hemocytes exposed to *Vibrio splendidus* LGP32 strain: A transcriptomic attempt at identifying molecular actors. Fish Shellfish Immunol..

[55] Prasopdee S., Sotillo J., Tesana S., Laha T., Kulsantiwong J., Nolan M.J., Loukas A., Cantacessi C. (2014). RNA-Seq reveals infection-induced gene expression changes in the snail intermediate host of the carcinogenic liver fluke, *Opisthorchis viverrini*. PLoS Negl. Trop. Dis..

[56] Mastellos D., Lambris J.D. (2002). Complement: More than a ‘guard’ against invading pathogens?. Trends Immunol..

[57] Volanakis J.E. (1990). Participation of C3 and its ligands in complement activation. Curr. Top. Microbiol. Immunol..

[58] Dong Y., Sun H., Zhou Z., Yang A., Chen Z., Guan X., Gao S., Wang B., Jiang B., Jiang J. (2014). Expression analysis of immune related genes identified from the coelomocytes of sea cucumber (*Apostichopus japonicus*) in response to LPS challenge. Int. J. Mol. Sci..

[59] Yang A., Zhou Z., Pan Y., Jiang J., Dong Y., Guan X., Sun H., Gao S., Chen Z. (2016). RNA sequencing analysis to capture the transcriptome landscape during skin ulceration syndrome progression in sea cucumber *Apostichopus japonicus*. BMC Genom..

[60] Nauta A.J., Daha M.R., Tijsma O., van de Water B., Tedesco F., Roos A. (2002). The membrane attack complex of complement induces caspase activation and apoptosis. Eur. J. Immunol..

[61] Yang A., Zhou Z., Dong Y., Jiang B., Wang X., Chen Z., Guan X., Wang B., Sun D. (2010). Expression of immune-related genes in embryos and larvae of sea cucumber *Apostichopus japonicus*. Fish Shellfish Immunol..

[62] Barat A., Sahoo P.K., Kumar R., Goel C., Singh A.K. (2016). Transcriptional response to heat shock in liver of snow trout (*Schizothorax richardsonii*)—A vulnerable Himalayan Cyprinid fish. Funct. Integr. Genom..

[63] Zhang L.B., Feng Q.M., SUn L.N., Ding K., Huo D., Yan F., Zhang T., Yang H.S. (2018). Differential gene expression in the intestine of sea cucumber (*Apostichopus japonicus*) under low and high salinity conditions. Comp. Biochem. Physiol. Part D Genom. Proteom..

[64] Cao Y., Ohwatari N., Matsumoto T., Kosaka M., Ohtsuru A., Yamashita S. (1999). TGF-beta1 mediates 70-kDa heat shock protein induction due to ultraviolet irradiation in human skin fibroblasts. Eur. J. Physiol..

[65] Shao Y., Li C., Chen X., Zhang P., Li Y., Li T., Jiang J. (2015). Metabolomic responses of sea cucumber *Apostichopus japonicus* to thermal stresses. Aquaculture.

[66] Roberts R.J., Agius C., Saliba C., Bossier P., Sung Y.Y. (2010). Heat shock proteins (chaperones) in fish and shellfish and their potential role in relation to fish health: A review. J. Fish Dis..

[67] Nelson R.J., Ziegelhoffer T., Nicolet C., Werner-Washburne M., Craig E.A. (1992). The translation machinery and 70 kd heat shock protein cooperate in protein synthesis. Cell.

[68] Shen L., Song Y., Fu Y., Li P. (2018). MiR-29b mimics promotes cell apoptosis of smooth muscle cells via targeting on MMP-2. Cytotechnol.

[69] Ren C., Chen T., Sun H., Jiang X., Hu C., Qian J., Wang Y. (2015). The first echinoderm poly-U-binding factor 60 kDa (PUF60) from sea cucumber (*Stichopus monotuberculatus*): Molecular characterization, inducible expression and involvement of apoptosis. Fish Shellfish Immunol..

[70] Lee G.H., Kim H.K., Chae S.W., Kim D.S., Ha K.C., Cuddy M., Kress C., Reed J.C., Kim H.R., Chae H.J. (2007). Bax inhibitor-1 regulates endoplasmic reticulum stress-associated reactive oxygen species and heme oxygenase-1 expression. J. Biol. Chem..

[71] Reinehr R., Becker S., Wettstein M., Haussinger D. (2004). Involvement of the Src family kinase yes in bile salt-induced apoptosis. Gastroenterol.

[72] Liu S., Wang X., Sun F., Zhang J., Feng J., Liu H., Rajendran K.V., Sun L., Zhang Y., Jiang Y. (2013). RNA-seq reveals expression signatures of genes involved in oxygen transport, protein synthesis, folding, and degradation in response to heat stress in catfish. Physiol. Genom..

[73] Artigaud S., Richard J., Thorne M.A., Lavaud R., Flye-Sainte-Marie J., Jean F., Peck L.S., Clark M.S., Pichereau V. (2015). Deciphering the molecular adaptation of the king scallop (*Pecten maximus*) to heat stress using transcriptomics and proteomics. BMC Genom..

[74] Polato N.R., Voolstra C.R., Schnetzer J., DeSalvo M.K., Randall C.J., Szmant A.M., Medina M., Baums I.B. (2010). Location-specific responses to thermal stress in larvae of the reef-building coral *Montastraea faveolata*. PLoS ONE.

[75] Gallagher P.J., Herring B.P., Stull J.T. (1997). Myosin light chain kinase. J. Muscle Res. Cell Motil..

[76] Farah M.E., Sirotkin V., Haarer B., Kakhniashvili D., Amberg D.C. (2011). Diverse protective roles of the actin cytoskeleton during oxidative stress. Cytoskeleton.

[77] Larsen M., Artym V.V., Green J.A., Yamada K.M. (2006). The matrix reorganized: Extracellular matrix remodeling and integrin signaling. Curr. Opin. Cell Biol..

[78] NCBI Sequence Read Archive. https://trace.ncbi.nlm.nih.gov/Traces/sra/sra.cgi?.

[79] Gene Ontology Database. http://www.geneontology.org/.

[80] Kyoto Encyclopedia of Genes and Genomes Database. http://www.genome.jp/kegg/.

